# A detection method for latent circadian rhythm sleep-wake disorder

**DOI:** 10.1016/j.ebiom.2020.103080

**Published:** 2020-11-06

**Authors:** Makoto Akashi, Reimi Sogawa, Ritsuko Matsumura, Atsuhiro Nishida, Rino Nakamura, Isao T. Tokuda, Koichi Node

**Affiliations:** a The Research Institute for Time Studies, Yamaguchi University, 1677-1 Yoshida, Yamaguchi 753-8511, Yamaguchi, Japan; b Department of Clinical Genetics and Genomic Medicine, Okayama University Hospital, 2-5-1 Shikata-cho, Kitaku, Okayama, Japan; c Department of Mechanical Engineering, Ritsumeikan University, 1-1-1 Nojihigashi, Kusatsu, Japan; d Department of Cardiovascular Medicine, Saga University, 5-1-1 Nabeshima, Saga, Japan

**Keywords:** Circadian rhythm sleep-wake disorder, Circadian clock, Hair follicle, Clock gene, *Period3*

## Abstract

**Background:**

Individuals with typical circadian rhythm sleep-wake disorders (CRSWDs) have a habitual sleep timing that is desynchronized from social time schedules. However, it is possible to willfully force synchronisation against circadian-driven sleepiness, which causes other sleep problems. This pathology is distinguishable from typical CRSWDs and is referred to here as latent CRSWD (LCRSWD). Conventional diagnostic methods for typical CRSWDs are insufficient for detecting LCRSWD because sufferers have an apparently normal habitual sleep timing.

**Methods:**

We first evaluated the reliability of circadian phase estimation based on clock gene expression using hair follicles collected at three time points without sleep interruption. Next, to identify detection criteria for LCRSWD, we compared circadian and sleep parameters according to estimated circadian phases, at the group and individual level, between subjects with low and high Pittsburgh Sleep Quality Index (PSQI) scores. To validate the reliability of identified detection criteria, we investigated whether the same subjects could be reproducibly identified at a later date and whether circadian amelioration resulted in sleep improvement.

**Findings:**

We successfully validated the reliability of circadian phase estimation at three time points and identified potential detection criteria for individuals with LCRSWD attributed to delayed circadian-driven sleepiness. In particular, a criterion based on the interval between the times of the estimated circadian phase of clock gene expression and getting out of bed on work or school days was promising. We also successfully confirmed the reproducibility of candidate screening and sleep improvement by circadian amelioration, supporting the reliability of the detection criteria.

**Interpretation:**

Although several limitations remain, our present study demonstrates a promising prototype of a detection method for LCRSWD attributed to delayed circadian-driven sleepiness. More extensive trials are needed to further validate this method.

**Funding:**

This study was supported mainly by JSPS, Japan.

**Research in Context**

**Evidence before this study**

Sleep disorders attributed to circadian dysfunction are called circadian rhythm sleep-wake disorders (CRSWDs). Individuals with typical CRSWDs have a habitual sleep timing that is desynchronized from social time schedules. However, it is possible to willfully force synchronisation against circadian-driven sleepiness, which causes other sleep problems. This type of CRSWD is referred to here as latent CRSWD (LCRSWD). Given that one-third of the European population suffers from 2 h or more of social jetlag, the number of people suffering from LCRSWD could be substantial. Nevertheless, conventional diagnostic methods for typical CRSWDs are insufficient for detecting LCRSWD because sufferers have an apparently normal habitual sleep timing.

**Added value of this study**

In this study, we compared circadian and sleep parameters according to estimated circadian phases between subjects with and without sleep difficulties and identified potential detection criteria for LCRSWD. Furthermore, we evaluated the reliability of these detection criteria through follow-up investigations and successfully confirmed the reproducibility of candidate screening and sleep improvement by circadian amelioration. Our present study therefore demonstrates a promising prototype of a detection method for LCRSWD.

**Implications of all the available evidence**

The number of people potentially suffering from LCRSWD is likely considerable, particularly among younger generations. Although circadian amelioration is a causal treatment for patients with LCRSWD, these patients might be prescribed general symptomatic treatments for sleep problems due to the lack of a well-established diagnostic method for LCRSWD. Our detection method will contribute to realizing more reliable diagnosis of LCRSWD for proper medical treatment.

Alt-text: Unlabelled box

## 1 Introduction

Sleep disorders are caused by a wide range of internal and external factors [1], [2], [3], [4], [5], indicating the need for reliable identification of specific causes in a personalized manner for proper medical treatment. Sleep disorders attributed to circadian dysfunction are called circadian rhythm sleep-wake disorders (CRSWDs), which are subdivided into several disorders: advanced sleep-wake phase disorder, delayed sleep-wake phase disorder, non-24 h sleep-wake rhythm disorder and irregular sleep-wake rhythm disorder [6], [7], [8], [9], [10]. These typical CRSWDs have been reported to affect approximately 1% to 3% of the population [11], [12], [13], [14]. Patients suffering from CRSWDs are characterized by the desynchrony in their habitual sleep timing from social time schedules. Diagnosis of CRSWDs is therefore relatively easy because the desynchrony from social time schedules is simply and clearly detectable using sleep diaries and actograms [15], [16], [17]. In the clinical treatment of patients diagnosed with CRSWDs, γ-aminobutyric acid (GABA) receptor agonists and orexin receptor antagonists may be counterproductive. Instead, the circadian clock should be adjusted using bright light and/or melatonin receptor agonists to help synchronize the patient's circadian-driven sleepiness with social time schedules [8,18,19].

**Fig. 1:**
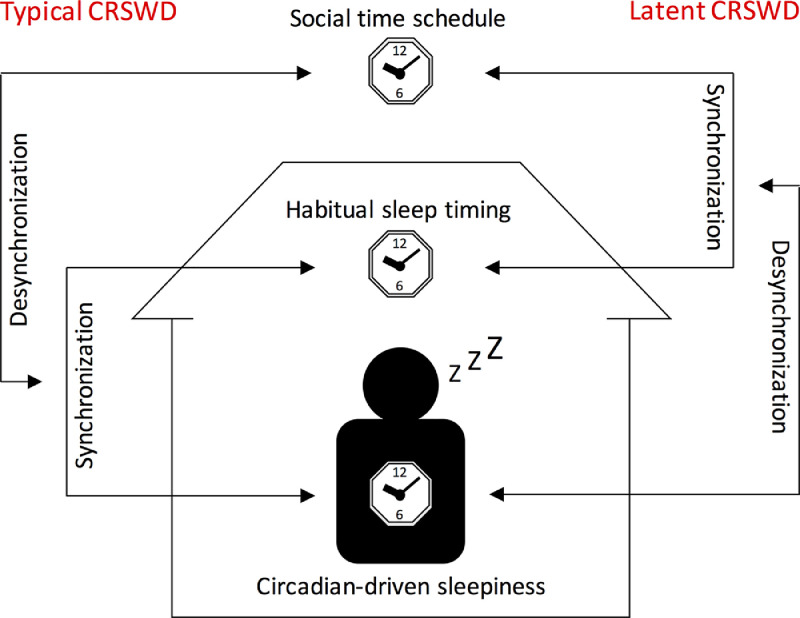
Typical circadian rhythm sleep-wake disorders (CRSWDs) and latent CRSWD (LCRSWD). In individuals with normal sleep, the habitual sleep timing is synchronized with social time schedules and circadian-driven sleepiness (*i.e.*, sleepiness independent of homeostatic sleep pressure but under the control of the circadian clock). While patients with typical CRSWDs have a habitual sleep timing that is synchronized with circadian-driven sleepiness but not with social time schedules, patients with LCRSWD have a habitual sleep timing that is synchronized with social time schedules but not with circadian-driven sleepiness.

Patients suffering from typical CRSWDs, as described above, have a habitual sleep timing that is desynchronized from social time schedules due to abnormal circadian-driven sleepiness. However, it is possible for people to force their own habitual sleep timing to synchronize with social time schedules against circadian-driven sleepiness. In this case, the misalignment between circadian-driven sleepiness and habitual sleep timing that is synchronized with social time schedules causes other sleep problems. Given that the patient's apparent habitual sleep timing is normal, this type of CRSWD is distinguishable from typical CRSWDs. Although the third edition of the International Classification of Sleep Disorders describes this disease state as one of the pathologies of CRSWDs [10], there remains no specific definition or terminology. In the present study, we describe this type of CRSWD as latent CRSWD (LCRSWD) (Fig. 1). While patients with typical CRSWDs have a habitual sleep timing that is synchronized with circadian-driven sleepiness but not with social time schedules, patients with LCRSWD have a habitual sleep timing that is synchronized with social time schedules but not with circadian-driven sleepiness. Given modern environments and lifestyle habits, the number of people potentially suffering from LCRSWD may be substantial, particularly among younger generations. Social jetlag, which is calculated based on the difference between the midpoints of sleep on work days and days off (without alarm clocks), is an often-used index to quantify the discrepancy between circadian and social clocks. A large-scale survey of European participants revealed that one-third of the population suffers from 2 h or more of social jetlag [20]. Because social jetlag does not necessarily but can potentially cause circadian-related sleep problems, this epidemiological study on social jetlag strongly indicates that the number of people suffering from LCRSWD is considerable, particularly among younger generations. In the treatment of patients with LCRSWD, as for typical CRSWDs, bright light exposure and/or administration of melatonin receptor agonists help synchronize circadian-driven sleepiness with habitual sleep timing and social time schedules.

Administration of proper medical treatment to patients with LCRSWD requires the establishment of a reliable diagnostic method for LCRSWD. While sleep parameters such as awakening and bedtime hours obtained using sleep diaries and actograms are powerful for diagnosing typical CRSWDs, they are insufficient for detecting LCRSWD because the habitual sleep timing of patients is synchronized with social time schedules. However, the habitual sleep timing on days off independent of social time schedules likely reflects the phase of circadian-driven sleepiness [20]. Therefore, a larger difference in the habitual sleep timing between work days and days off is more likely to indicate LCRSWD. On this basis, comparison of awakening and bedtime hours between work days and days off is a helpful initial evaluation of an individual's potential risk of LCRSWD. However, it remains unclear which parameters related to habitual sleep timing are more direct indicators of LCRSWD.

Reliable diagnosis of LCRSWD requires biological markers because while the habitual sleep timing on days off reflects the phase of circadian-driven sleepiness, it can be affected by various non-circadian factors, particularly sleep debt. The circadian clock defines the phase of circadian-driven sleepiness [21,22]. Comparison of the circadian clock phase estimated using biological markers with the habitual sleep timing may therefore enable evaluation of the potential risk of LCRSWD, with abnormal intervals between these phases indicating misalignment between circadian-driven sleepiness and the habitual sleep timing. Although melatonin is thought to be a reliable biological marker for the circadian clock phase in a research setting [6,7,23], it is associated with several limitations: the functional role of endogenous melatonin in the circadian oscillator is unclear [24,25], and subjects must remain under dim light conditions during sample collection because melatonin levels are decreased in response to light input [26]. Although a reported method using metabolites is powerful for circadian phase estimation and can overcome limitations in melatonin measurement [27], application of this method in clinical settings requires overcoming the cost barrier. Circadian clock phase estimation based on peripheral clock gene expression may therefore be an alternative option for experimental evaluation of the potential risk of LCRSWD.

In the present study, we first evaluated the reliability of circadian phase estimation based on clock gene expression using hair follicles collected at three time points. Next, we aimed to identify potential detection criteria for individuals with LCRSWD by examining circadian and sleep parameters based on the estimated circadian phases. To validate the identified detection criteria, we investigated the reproducibility of subject screening and the effect of circadian amelioration on sleep.

## 2 Methods

### 2.1 Ethics statement

This study was conducted in accordance with the Declaration of Helsinki and all procedures were approved by the institutional review boards of Yamaguchi University and Saga University, with all subjects providing written informed consent.

### 2.2 Subjects

A total of 24 subjects (9 males and 15 females) with a Pittsburgh Sleep Quality Index (PSQI) score ≤ 5 were defined as the control group, and 23 subjects (8 males and 15 females) with a PSQI score > 5 were assigned to the H-PSQI group. Because all subjects were able to adapt themselves to social time schedules during all experimental periods, none of them had typical CRSWD. The subjects in the two groups had comparable age, body mass index (BMI) and smoking status, as shown in Table 1. No subjects in either group took sleep medication. The Japanese version of the Morningness-Eveningness Questionnaire (MEQ) was used to assess the chronotype of all subjects [28]. In addition, all subjects completed the Japanese version of the Munich Chronotype Questionnaire (MCTQ), which is a powerful tool for obtaining well-defined sleep parameters such as GUw, local time of getting out of bed on work or school days; SOw, sleep onset on work or school days; GUf, local time of getting out of bed on free days; and SOf, sleep onset on free days [29].

**Table 1:** Demographic, sleep and circadian characteristics of groups with low and high PSQI scores.

Characteristic	Control Group (PSQI ≤ 5) *n* = 24	High PSQI Group (PSQI > 5) *n* = 23	*P* Value^a^^,^^b^
Sex, male/female	9/15	8/15	0.23 (0.37)
Age, y (SD)	23.17 (5.86)	21.13 (1.25)	0.11 (0.29)
BMI (SD)	20.19 (2.19)	21.14 (2.72)	0.19 (0.37)
Overweight (BMI: 25.0–29.9), No.	1	1	0.51 (0.58)
Sleep medication, yes/no	0/24	0/23	1.0 (1.0)
Smoking status, yes/no	2/22	1/22	0.39 (0.52)
PSQI score (SD)	2.71 (1.55)	11.26 (3.25)	< 0.001 (< 0.001)
MEQ score (SD)	54.25 (8.26)	41.7 (8.96)	< 0.001 (< 0.001)
Morning type, No.	8	2	
Neither type, No.	14	10	
Evening type, No.	2	11	

Abbreviations: BMI, Body Mass Index; PSQI, Pittsburgh Sleep Quality Index; MEQ, Morningness-Eveningness Questionnaire.

a Statistical comparisons of groups with low and high PSQI scores were performed using the unpaired two-tailed Student's t-test or Fisher's exact test.

b The method of Benjamini-Hochberg was used to calculate adjusted P values (in parentheses).

### 2.3 Experimental design

In contrast to typical CRSWDs, subjects with LCRSWD should be classified under the H-PSQI group because the misalignment between circadian-driven sleepiness and habitual sleep timing on work days causes quantitative and qualitative sleep problems. Based on an epidemiological study on social jetlag [20], we hypothesized that LCRSWD is one of the major contributors to sleep problems, particularly among younger generations. Therefore, we first compared circadian and sleep parameters between the control and H-PSQI groups to identify detection criteria for LCRSWD. Although sleep problems are caused by a wide range of internal and external factors in a combinatory manner, the population-level contribution of circadian dysfunction to sleep problems can be evaluated through statistical comparison of these parameters between the control and H-PSQI groups. Circadian-related parameters showing a major difference between the groups are expected to be promising candidate detection criteria for LCRSWD. Following these statistical intergroup comparisons, we next conducted interindividual comparisons of these parameters. Subjects completed the PSQI, MEQ and MCTQ, and had scalp hair follicles collected at three time points (right after awakening, in the afternoon and right before bedtime) at approximately 8‐hr intervals around the clock without sleep interruption.

Based on details of informed consent, all subjects were provided a brief summary of the experimental results. In response to this information, subjects identified as potentially having LCRSWD decided themselves to try a well-known general method for circadian amelioration. Subjects with LCRSWD should show improved sleep following circadian amelioration because their sleep problems are largely attributed to circadian dysfunction. We therefore continued observational experiments to evaluate whether we could detect LCRSWD based on our criteria. Based on our expert advice, the subjects followed the three rules described below for as many days as possible to ameliorate their delayed circadian-driven sleepiness. First, subjects exposed themselves to sunlight for more than 30 min in the morning. They were informed that this sunlight exposure should be done within 2 h of awakening. Second, subjects refrained from using light-emitting devices such as smartphones and computers two hours before bedtime. Third, subjects reduced the time-lag of morning awakening between work/school and free days. To avoid inducing excessive mental and physical stress, we recommended that subjects reduce their sleep debt by having an earlier bedtime on free days. These three rules for circadian amelioration were performed for a period of more than three weeks and continued for as long as possible based on the subject's decision according to their mental and physical conditions.

### 2.4 Peripheral clock gene expression

To identify the optimal time interval for sample collection at three time points, we performed circadian phase estimation with various time intervals using a cosine curve fitted to actual measurements obtained for healthy volunteers. We found that the accuracy of circadian phase estimation was highest at a time interval of about 8 h. Scalp hair follicles were collected by gripping and tugging the hair shaft with a pair of tweezers. The hair shafts, which were at least partially covered with hair follicle cells, were quickly soaked in cell lysis buffer (RNAqueous-Micro Kit; Thermo Fisher Scientific, U.S.A.). Hair follicle cells attached to the hair shafts were stored at below −20 °C until total RNA purification. Approximately 2–10 scalp hair follicles were required at each time point to stably and reproducibly detect clock gene expression. To minimize skin damage, hair follicles were collected from different regions of the scalp. After mild homogenisation by hand and removal of the hair shaft, the RNAqueous-Micro Kit was used to purify total RNA. After checking the quality and concentration using a NanoDrop (LMS, Japan), total RNA was reverse-transcribed using a SuperScript VILO cDNA Synthesis Kit (Life Technologies, U.S.A.), and real-time PCR was performed using a TaqMan MGB probe (Applied Biosystems, U.S.A.) and a 1/20-volume of the reverse transcription product. Data were obtained using PRISM7300 (Applied Biosystems, U.S.A.) and normalised against *18S ribosomal RNA* (*18S-rRNA*), the expression of which is constant regardless of cell type and sampling time [30]. Primer and probe sequences of the *Period 3* (*Per3*), *Nr1d1* (*nuclear receptor subfamily 1 group D member 1*, also known as *Rev-erbα*), *Nr1d2* (*nuclear receptor subfamily 1 group D member 2*, also known as *Rev-erbβ*) and *18S-rRNA* transcripts are listed in our previous report [31].

### 2.5 Circadian phase estimation based on expression data from two clock genes at three time points

The circadian clock, which is driven by autonomous transcriptional oscillation of clock genes, defines the phase of circadian-driven sleepiness. Because the phase intervals among clock gene transcripts are similar regardless of cell type [30], any clock gene transcript can be used as a phase marker for circadian-driven sleepiness. The peak time of *Per3* transcripts (*Per3* peak time, P3PT) was used as the phase marker in the present study. The method used to estimate P3PT is described below.

First, the time-course of gene expression was normalized to fit to 24-h-period cosine curves with unity amplitude. The normalized gene expression at time t was denoted *x*(*t*) and *y*(*t*) for *Per3* and *Nr1d2*, respectively. Second, gene expression data from the two genes were fitted to the following 24-h-period cosine curves under the constraint that *Per3* and *Nr1d2* had a phase difference of Δθ:

$$x\left(t\right)=A_{x}\cos\left(\omega t+\theta \right)+C_{x}$$

$$y\left(t\right)=A_{y}\cos\left(\omega t+q+\Delta q\right)+C_{y}$$

For measurements conducted at three time points, the cost function was defined as

$$E\left(\theta ,A,C\right)=\sum_{i=1}^{3}\left[{\left\{x\left(t_{i}\right)-A_{x}\cos\left(\omega t_{i}+\theta \right)-C_{x}\right\}}^{2}+{\left\{y\left(t_{i}\right)-A_{y}\cos\left(\omega t_{i}+\theta +\Delta \theta \right)-C_{y}\right\}}^{2}\right],$$

where ω=2π/24. The model parameters, $\left\{\theta ,A_{x},A_{y},C_{x},C_{y}\right\}$, were determined so as to minimize the cost function using the conjugate gradient method. Empirically, the difference in peak expression time between the two clock genes is known to be around Δt=2h, which is translated to the phase difference of Δθ=2πΔτ/24. The optimal phase difference, which gives the minimum cost function, was selected by varying the phase difference in the range Δτ [1.5 h, 2.5 h]. Finally, the peak expression time of *Per3* was estimated to be CT_Per3_ = 24(0.5π−θ)/(2π). To evaluate goodness of fit, the coefficient of determination was also calculated from the three-point data.

### 2.6 Statistical analysis

No statistical methods were used to predetermine the sample size due to the lack of available prevalence estimation for LCRSWD. Statistical comparisons of groups with low and high PSQI scores were performed using the unpaired two-tailed Student's t-test or Fisher's exact test. The Benjamini-Hochberg method was used to calculate adjusted P values. Linear correlation between estimated circadian phases and sleep parameters was evaluated using Pearson correlation. *P* < 0.05 indicated statistical significance. Receiver Operating Characteristic (ROC) analysis was performed using MATLAB (R2020a, MathWorks).

### 2.7 Role of funding source

The funders had no role in the study design, data collection, data analysis, data interpretation, writing of the report or the decision to publish.

## 3 Results

### 3.1 Validation of circadian phase estimation based on expression of two clock genes at three time points

**Fig. 2:**
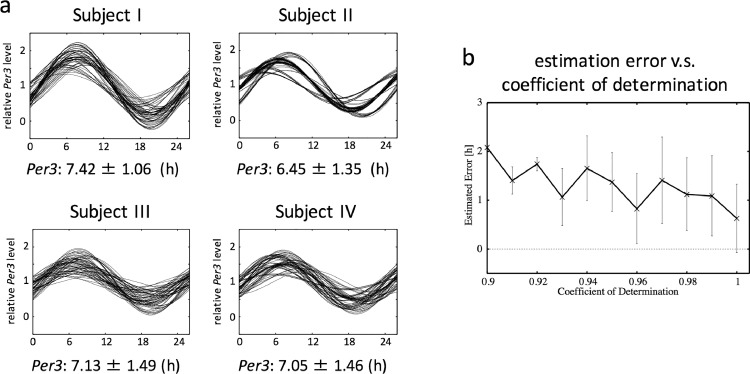
Validation of circadian phase estimation based on expression of two clock genes at three time points. Scalp hair follicles were collected from four subjects at three time points at approximately 8‐h intervals without sleep interruption. Four independent samples were collected at each time point to perform accuracy validation of circadian phase estimation. Total RNA was subjected to real‐time PCR for quantification of the expression levels of *Per3* and *Nr1d2*. Expression levels were normalized based on the expression level of *18S ribosomal RNA*. (a) For four subjects (I, II, II and IV), 64 combinations of data from three time points were constructed from differently but simultaneously sampled data. Cosine curve fitting was performed for each combination of *Per3* and *Nr1d2* expression data, and the 64 fitted curves were then overlaid. Variability in the fitted curves represents the estimation error. The mean of 64 estimated circadian phases was defined as the real phase. The real phase ± standard deviation is shown for each subject. (b) Dependence of the estimation error (*i.e.*, deviation of the estimated phase from the mean phase) on the coefficient of determination of cosine fitting.

We validated the reliability of circadian phase estimation based on expression data from two clock genes (*Per3* and *Nr1d2*) at three time points using sample data sets obtained from healthy subjects (Fig. 2a). Scalp hair follicle cells were collected from four subjects at three time points at approximately eight-hour intervals without sleep interruption. Four independent samples were collected at each time point to perform accuracy validation of circadian phase estimation. Because the phase interval between *Per3* and *Nr1d2* circadian expression is mostly constant among individuals [31], cosine fitting was conducted under the condition that the *Per3* and *Nr1d2* phase interval was within a range of 2 ± 0.5 h. Circadian phase (*i.e.* P3PT) was estimated in 64 combinations because we had four independent samples for each time point, and we defined the mean of all 64 estimated circadian phases as the real phase. The estimation error and coefficient of determination (CD) were obtained from the 64 estimations for each subject (Fig. 2b). Investigation of the relationship between the estimation error and CD revealed that the mean error was about one hour when CD was greater than 95%.

### 3.2 Search for detection criteria for LCRSWD through statistical comparison of groups with low and high PSQI scores

To identify variables specific to LCRSWD, sleep and circadian parameters were statistically compared between groups with low (PSQI ≤ 5, control group) and high PSQI scores (PSQI > 5, H-PSQI group) (Tables 1 and 2). The H-PSQI group had a significantly lower MEQ score than the control group (Table 1). SOw, GUf and SOf were significantly delayed by an average of about one hour in the H-PSQI group (Table 2). SOw − P3PT was significantly larger in the H-PSQI group. Although we validated the estimation of P3PT using expression data from two genes (2 G, *Per3* and *Nr1d2*) at three time points in Fig. 2, for further confirmation, we reproduced all estimates using expression data from three genes (3 G, *Per3, Nr1d1* and *Nr1d2*). These results provided no potential detection criteria for LCRSWD.

**Table 2:** Statistical comparison of sleep and circadian parameters between groups with low and high PSQI scores.

Characteristic	Control Group (PSQI ≤ 5) *n* = 24		High PSQI Group (PSQI > 5) *n* = 23		*P* Value^a^
Mean	SD		Mean	SD	
Working or school day					
GUw (local time)	7.99	0.81	8.45	1.14	0.111
SOw (local time)	24.52	0.79	25.78	1.01	0.000*
Free day					
GUf (local time)	9.16	1.22	10.07	1.55	0.031*
SOf (local time)	25.05	1.07	26.03	1.21	0.005*
Time lag					
GUf − GUw (h)	1.17	0.89	1.61	1.29	0.178
SOf − SOw (h)	0.52	0.75	0.25	0.92	0.277
*Per3* peak time (P3PT)					
2 G (local time)	6.40	1.14	6.57	0.82	0.564
3 G (local time)	6.45	1.32	6.68	0.93	0.497
Time interval (2 G)					
GUw − P3PT (h)	1.59	1.16	1.89	1.48	0.445
SOw − P3PT (h)	18.12	1.16	19.21	0.99	0.001*
GUf − P3PT (h)	2.76	1.56	3.50	1.78	0.138
SOf − P3PT (h)	18.65	1.50	19.47	1.42	0.061
Time interval (3 G)					
GUw − P3PT (h)	1.54	1.28	1.77	1.52	0.564
SOw − P3PT (h)	18.07	1.26	19.10	0.94	0.003*
GUf − P3PT (h)	2.71	1.58	3.39	1.84	0.182
SOf − P3PT (h)	18.60	1.56	19.35	1.45	0.092

Abbreviations: GUw, local time of getting out of bed on working or school days; SOw, sleep onset on working or school days; GUf, local time of getting out of bed on free days; SOf, sleep onset on free days; 2 G, local time of *Per3* peak time estimated using three-point expression data of two genes, *Per3* and *Nr1d2*; 3 G, local time of *Per3* peak time estimated using three-point expression data of three genes, *Per3, Nr1d1* and *Nr1d2*.

a Statistical comparisons of groups with low and high PSQI scores were performed using the unpaired two-tailed Student's *t*-test.

Linear correlation analysis was performed between estimated circadian phases and sleep parameters (Supplementary Figure 1). According to two-dimensional scatter plots (P3PT *versus* MEQ, GUw, SOw, GUf or SOf), while there was a moderate positive correlation (*r* > 0.3) between P3PT and GUw in the control group, there was no such correlation in the H-PSQI group. A moderate positive correlation was observed between P3PT and SOw in both groups. Despite SOw being significantly delayed in the H-PSQI group (Table 2), the level of correlation between P3PT and SOw was similar to that in the control group. These results suggest that the relationship between GUw and P3PT may define the detection criteria for LCRSWD. However, due to the large confidence intervals (CIs), the present sample size was insufficient to make any robust interpretations.

### 3.3 Identification of detection criteria for LCRSWD through interindividual comparison between groups with low and high PSQI scores

**Fig. 3:**
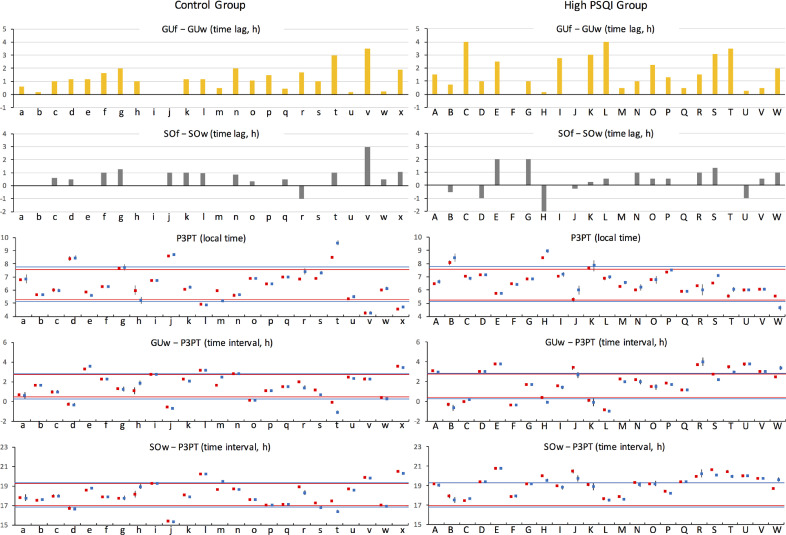
Interindividual comparison of sleep and circadian parameters between groups with low and high PSQI scores. The first and second panels from the top show GUf – GUw (yellow bars) and SOf – SOw (gray bars) for each subject, respectively. The dot plots show the value of P3PT, GUw − P3PT and SOw − P3PT with 95% CI for each subject (red dots, P3PT estimated from 2 genes; blue dots, P3PT estimated from 3 genes). To facilitate visual identification of potential abnormalities, the 1 SD range of values from the control group was indicated on every dot plot for both the control and H-PQSI groups (top line, mean + SD; bottom line, mean − SD). For example, the top and bottom lines shown in the P3PT plot for the control group, which indicate a 1 SD range of values for P3PT in the control group, are also shown in the P3PT plot for the H-PSQI group. Red or blue lines indicate that the 1 SD range was calculated using P3PT data estimated from 2 or 3 genes, respectively. (For interpretation of the references to color in this figure legend, the reader is referred to the web version of this article.)

Circadian and sleep parameters were compared at the individual level between groups with low and high PSQI scores (Fig. 3). In both groups, many subjects showed larger GUf − GUw than SOf − SOw. Although there was no significant difference in GUf − GUw between the groups (Table 2), two subjects in the control group had a GUf − GUw of more than two hours compared to eight subjects in the H-PSQI group. In particular, Subjects C and L showed a GUf − GUw of four hours. To facilitate visual identification of potential abnormalities, the 1 SD range of values from the control group is shown on every dot plot in Fig. 3 (top line, mean + SD; bottom line, mean − SD). Although the distribution of P3PT in the H-PSQI group was similar to that in the control group, the GUw − P3PT value showed larger individual differences in the H-PSQI group. Subjects C and L had a GUw − P3PT value less than the mean – SD of the control group. Nearly half of the H-PSQI subjects had a SOw − P3PT greater than the mean + SD of the control group. According to these results, two criteria were set for LCRSWD due to delayed circadian-driven sleepiness: a GUf − GUw of about three hours or more and a GUw − P3PT less than the mean – SD of the control group. Subjects C, K and L met both criteria.

### 3.4 Reproducibility of candidate screening

**Fig. 4:**
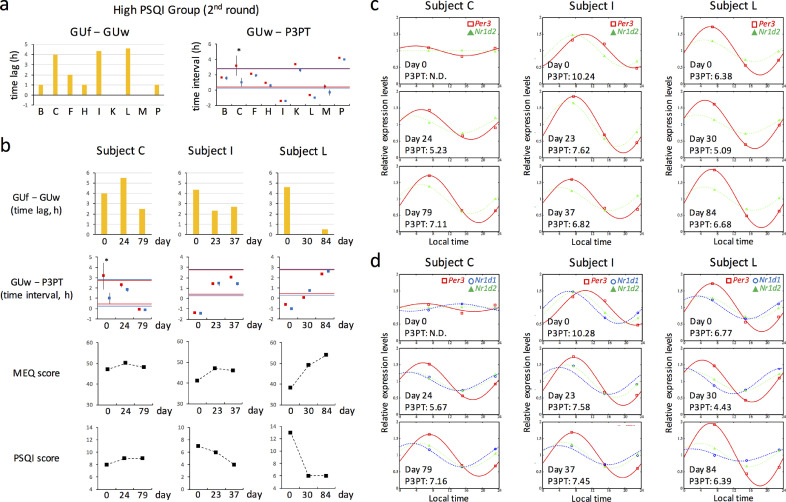
Validation of detection criteria for LCRSWD through follow-up investigations (a) About two months after the assessment shown in Fig. 3, a second assessment was conducted for H-PSQI subjects with GUf − GUw > 2 h or GUw − P3PT < the mean – SD of the control group in the first assessment. Subjects M and P, who met neither of the criteria in the first assessment, were recruited as negative control subjects. (b) Subjects C, I and L performed circadian amelioration for a period of several weeks (C, 24 days; I, 23 days; L, 30 days). They continued to perform circadian amelioration for as long as possible (C, 79 days; I, 37 days; L, 84 days, in total). (a and b) GUf – GUw (yellow bars) and GUw − P3PT with 95% CI (dot plots) are shown for each subject. Red and blue dots indicate that P3PT was estimated based on expression of two and three clock genes, respectively. The 1 SD range of GUw − P3PT for the control group in Fig. 3 is again shown on these dot plots (red lines, estimation from 2 genes; blue lines, estimation from 3 genes). (c and d) Clock gene expression rhythms of Subjects C, I and L before (day 0) and after circadian amelioration. A mathematical estimation of P3PT was performed based on the expression levels of two (c) or three clock genes (d). Coloured curves and dots represent estimated and experimental values, respectively. Estimated P3PT values are shown below the curves (N.D., not determined). (For interpretation of the references to color in this figure legend, the reader is referred to the web version of this article.)

After about two months, a second screen was conducted for H-PSQI subjects with a GUf − GUw of more than two hours or GUw − P3PT less than the mean – SD of the control group in the first assessment. Seven H-PSQI subjects agreed to this second assessment. Subjects M and P, who had met neither of the criteria in the first assessment, were recruited as negative control subjects. All nine subjects again showed a high PSQI score (> 5). Compared to the first assessment, GUf − GUw decreased in Subject K, remained at about four hours in Subjects C and L and increased to more than four hours in Subject I (Fig. 4a, left). While GUw − P3PT improved in Subjects B, F, H and K in the second assessment, the value was reproducibly and newly less than the mean – SD of the control group in Subjects L and I, respectively (Fig. 4a, right). Subject C had severe circadian dysfunction because clock gene expression showed no circadian rhythms. Thus, Subjects C, I, and L were suspected of having LCRSWD.

To evaluate the accuracy of prediction of LCRSWD based on only a single criterion (GUw − P3PT or GUf − GUw), area under the curve (AUC) was calculated from ROC curves (Supplementary Figure 2a). The value of AUC was more than 0.9 for each criterion, indicating that the prediction of LCRSWD is highly reliable. The optimal cut-off value for single-criterion prediction was 0.15 for GUw − P3PT (2 G), 0.16 for GUw − P3PT (3 G) and 3 for GUf − GUw (Supplementary Figure 2b).

### 3.5 Effect of circadian amelioration on sleep

Based on our experimental results, in addition to Subjects C and L, Subject I, who showed obvious abnormalities in both criteria in the second assessment, underwent circadian amelioration by following the three rules detailed in the Methods section. We assessed their circadian and sleep parameters to validate our detection criteria. Three or four weeks later, these subjects completed the PSQI, MCTQ and MEQ and their hair follicles were collected at about eight hour intervals without sleep interruption. After these assessments, they continued to follow the rules for circadian amelioration for as long as possible. Compared to the first and second assessments, Subjects I and L had a shorter GUf – GUw value, a GUw − P3PT value within the 1 SD range of the control group and a lower PSQI score (Fig. 4b). While P3PT steadily advanced in Subject I, in Subject L, it had advanced four weeks after the start of circadian amelioration but subsequently reversed back to the original value (Fig. 4c and 4d). In Subject C, although transcriptional oscillation was restored, no improvement was observed in the PSQI score (Fig. 4b, 4c and 4d).

## 4 Discussion

### 4.1 Circadian phase estimation at three time points

Patients with typical CRSWDs have a habitual sleep timing that is synchronized with circadian-driven sleepiness but not with social time schedules. In contrast, patients with LCRSWD have a habitual sleep timing that is synchronized with social time schedules but not with circadian-driven sleepiness. Although both CRSWDs and LCRSWD are attributed to circadian dysfunction, differences in their pathological mechanism remain unclear. Our long-term goal is to establish a detection method for LCRSWD for use in clinical settings. In the present study, we aimed to create a prototype of this method.

To minimize burden on patients and medical workers, low sample collection frequency and a simple measurement procedure are required for circadian phase estimation using biological markers. Our previous reports demonstrated that scalp and facial hair follicle cells are useful for circadian phase estimation [31] and indicated that reliable circadian phase estimation was possible based on expression data from three clock genes (*Per3, Nr1d1* and *Nr1d2*) at four time points per day [32]. To realize sample collection without sleep interruption and further improve practicality, we validated the reliability of circadian phase estimation based on expression data from two clock genes at three time points (at approximately eight-hour intervals). The real phase (the mean of 64 estimated circadian phases) and standard deviation (SD) calculated for each subject suggested that circadian phase estimation based on the expression of two clock genes at three time points was practicable.

The mean error was about one hour when CD was greater than 95%. In subsequent experiments, about 5% of the data had a CD less than 95%. The value of CD is therefore a useful reliability indicator for circadian phase estimation. Lower CD values indicate lower reliability of estimated circadian phases, suggesting the existence of either technical issues related to the experimental procedure or a pathological sign due to circadian dysfunction.

### 4.2 Statistical search for detection criteria for LCRSWD

Consistent with MEQ scores, SOw, GUf and SOf were significantly delayed by an average of about one hour in the H-PSQI group. However, because a delayed sleep phase is not only caused by circadian dysfunction but also often results from a delayed habitual bedtime or difficulties with sleep onset, these parameters are not highly specific to LCRSWD. Although the H-PSQI group showed delayed GUw by an average of about 30 min, there was no statistically significant difference between groups. This is because GUw is strongly affected by social time schedules, such as the start time of business or class. In contrast, GUf is independent of social time schedules and is likely synchronized with circadian-driven sleepiness. The time lag between GUf and GUw is therefore a potential detection criterion for LCRSWD. However, although GUf − GUw in the H-PSQI group was an average of about 30 min longer, there was no statistically significant difference between groups. Thus, statistical comparison of these sleep timing parameters between the control and H-PSQI groups did not provide any potential detection criteria for LCRSWD.

Next, we statistically compared P3PT and its derivative parameters between groups. In LCRSWD, circadian-driven sleepiness and habitual sleep timing are desynchronous on work days. Habitual sleep timing on work days is defined by GUw and SOw, with the former being more affected by social time schedules and therefore likely to desynchronize from circadian-driven sleepiness. Because P3PT can be useful as a relative phase marker for circadian-driven sleepiness, the severity of the desynchrony between circadian-driven sleepiness and habitual sleep timing can therefore be evaluated based on the time interval between P3PT and GUw. However, there was no statistically significant difference in GUw − P3PT between groups. In contrast, SOw − P3PT was significantly larger in the H-PSQI group. As described above, delayed sleep onset may be independent of circadian dysfunction and SOw − P3PT cannot therefore be a specific criterion for LCRSWD.

Taken together, although we focused on GUf − GUw and GUw − P3PT as potential criteria for LCRSWD, there was no statistically significant difference between the control and H-PSQI groups. We speculate that statistical approaches were unable to identify detection criteria for LCRSWD because the contribution of LCRSWD to sleep problems in the H-PSQI group was unexpectedly minor.

### 4.3 Correlation between estimated circadian phases and sleep parameters

The sample size of the present study was insufficient to conduct correlation analysis and interpretation of the results of this analysis was limited due to the low statistical significance. However, the results provided a helpful indication of which among habitual sleep parameters affects circadian phase and what we should focus on to identify detection criteria for LCRSWD.

A moderate positive correlation between P3PT and GUw or SOw in the control group demonstrated that habitual sleep timing on work days affects P3PT. In contrast, there was lower or little correlation between P3PT and GUf or SOf. Given the ratio of free to work days per week, it makes sense that P3PT was more synchronous with sleep parameters on work days.

In contrast to the control group, there was no correlation between P3PT and GUw in the H-PSQI group. Although this result seems inconsistent with the lack of a significant difference in GUw, P3PT and GUw − P3PT between the control and H-PSQI groups, it likely resulted from the high individual differences in the time interval between P3PT and GUw in the H-PSQI group. In fact, the SD value of GUw − P3PT was larger in the H-PSQI group, suggesting the possibility that a proportion of the subjects in the H-PSQI group had LCRSWD due to desynchronisation between the habitual sleep timing and circadian-driven sleepiness.

These results suggest that interindividual, rather than intergroup, comparison of the time interval between GUw and P3PT may provide useful information for defining detection criteria for LCRSWD.

### 4.4 Identification of detection criteria for LCRSWD

To identify detection criteria for subjects with LCRSWD, who likely comprise a minority of the H-PSQI group, circadian and sleep parameters were compared at the individual level. Since GUw and GUf are likely affected by social time schedules and circadian-driven sleepiness, respectively, GUf − GUw should be strikingly long in subjects with LCRSWD. Based on this criterion, Subjects C and L showed a GUf − GUw of four hours, strongly suggesting that their circadian-driven sleepiness was abnormally delayed in comparison with social time schedules. They are therefore strong candidates for LCRSWD.

Although GUf − GUw may be a promising simple detection criterion for LCRSWD, this parameter can be affected by various non-circadian factors such as sleep debt. To further confirm LCRSWD, additional validation based on clock gene expression was indispensable. Interindividual comparison of P3PT and its derivative parameters between groups revealed that GUw − P3PT showed larger individual differences in the H-PSQI group, supporting our explanation for the loss of correlation between GUw and P3PT in this group. We therefore focused on H-PSQI subjects with a high or low GUw − P3PT value as potentially having LCRSWD.

Most of the H-PSQI subjects with a GUw − P3PT greater than the mean + SD of the control group also had a SOw − P3PT greater than the mean + SD of the control group. This indicates that their circadian-driven sleepiness was phase-advanced in comparison with their habitual sleep timing, which in turn suggests the presence of LCRSWD caused by advanced circadian-driven sleepiness. However, individuals with sleep problems often have a delayed habitual bedtime or difficulties with sleep onset independent of circadian dysfunction, which in turn delays sleep offset to maintain total sleep duration. It is therefore more plausible that, rather than their circadian-driven sleepiness being advanced, their SOw and GUw were delayed independently of circadian dysfunction. That is, the individual's circadian-driven sleepiness, as a result rather than a cause of sleep problems, was relatively advanced in comparison to their habitual sleep timing. Hence, LCRSWD is not likely to be a major contributor to sleep problems in H-PSQI subjects with a GUw − P3PT greater than the mean + SD of the control group.

In contrast, H-PSQI subjects with a GUw − P3PT less than the mean – SD of the control group, none of whom suffered from early-morning awakening, were thought to have LCRSWD because their circadian-driven sleepiness was delayed in comparison to their habitual sleep timing. Importantly, while Subjects C and L, strong candidates for LCRSWD based on GUf − GUw, had values inside the 1 SD range of the control group in P3PT, SOw − P3PT, GUf − P3PT and SOf − P3PT, they had a GUw − P3PT value less than the mean – SD of the control group.

Taken together, we concluded that individuals that met the following two criteria likely had LCRSWD due to delayed circadian-driven sleepiness: a GUf − GUw of about three hours or more and a GUw − P3PT less than the mean – SD of the control group. As an exception to the latter criterion, markedly low CD and/or large 95% CI in mathematical estimation of P3PT also indicates LCRSWD. However, in addition to Subjects C and L in the H-PSQI group, one subject in the control group also met these criteria. This suggests that meeting these criteria is required but not sufficient for the development of sleep problems. Therefore, additional factors, such as the chronicity of circadian misalignment, may be required for the development of LCRSWD.

### 4.5 Validation of detection criteria for LCRSWD

To further validate the reliability of our detection criteria for LCRSWD, we confirmed the reproducibility of candidate screening as well as sleep improvement by circadian amelioration through follow-up investigations.

If our detection criteria were appropriate, our candidate screen should reproducibly identify the same subjects as having LCRSWD at a later date. After about two months, a second screen based on the same criteria was therefore conducted on H-PSQI subjects. Compared to the first assessment, GUf − GUw remained at about four hours in Subjects C and L and increased to more than four hours in Subject I. GUw − P3PT was reproducibly and newly less than the mean – SD of the control group in Subjects L and I, respectively. Although GUw − P3PT appeared to improve in Subject C, surprisingly, clock gene expression remained mostly constant, as can be expected from a large 95% CI for GUw − P3PT. We have not encountered a subject with arrhythmic clock gene expression in any of our previous data using hair follicle cells from healthy and unhealthy subjects, strongly suggesting that Subject C had a severe circadian dysfunction. Even though Subjects B, F, H and K met neither of the criteria in the second assessment, their PSQI scores remained high, suggesting that circadian dysfunction may not be the main cause of sleep problems in these four subjects. Subjects C, K and L met both criteria for GUf – GUw and GUw − P3PT in the first assessment, and Subjects C, I and L met these criteria in the second assessment. Hence, our candidate screen reproducibly identified the same subjects (C and L) as having LCRSWD at a later date, and this reproducibility supports the reliability of our detection criteria for LCRSWD.

Subjects with LCRSWD who were successfully identified through our detection criteria were expected to show improved sleep following circadian amelioration because their sleep problems should be largely attributable to circadian dysfunction. Subjects C, I and L aimed to ameliorate circadian misalignment for three or four weeks. Circadian rhythms of Subjects I and L were considered to have been successfully adjusted because their GUf − GUw was shorter than in the first and second assessments and their GUw − P3PT was within the 1 SD range of the control group. As expected, they had a lower PSQI score. However, although P3PT had steadily advanced in Subject I, in Subject L, it had advanced four weeks after the start of circadian amelioration but subsequently reversed back to the original value. Nevertheless, sleep continued to improve in Subject L probably because GUw − P3PT remained within the 1 SD range due to a large delay in GUw. This confirms that the circadian phase relative to GUw, rather than the absolute circadian phase, is important for normal sleep. In contrast, no improvement was observed in the PSQI score in Subject C. Although clock gene expression in Subject C did not show clear circadian rhythms before the start of circadian amelioration, about three weeks later, transcriptional oscillation was restored and GUw − P3PT was within the normal range. The reason for the lack of sleep improvement is probably that GUf − GUw exceeded five hours. About eight weeks after the start of circadian amelioration, whereas GUf − GUw improved in Subject C, GUw − P3PT had reversed back to being less than the mean – SD of the control group. Hence, these results demonstrate that simultaneous improvement in GUf − GUw and GUw − P3PT is likely required for sleep improvement.

Taken together, these findings indicate that circadian amelioration resulted in sleep improvements in these subjects in the expected manner, again supporting the reliability of our detection criteria for LCRSWD.

## 5 Conclusions and limitations

The present study describes a successful prototype of a detection method for LCRSWD. Specifically, we demonstrated that a criterion based on GUw − P3PT is promising for the detection of LCRSWD caused by delayed circadian-driven sleepiness. As an exception, markedly low CD and/or large 95% CI in the estimation of P3PT also indicates LCRSWD. In addition, we demonstrated that GUf – GUw is useful as a simple initial criterion before proceeding to experimental evaluation of GUw − P3PT. Follow-up investigations conducted to confirm the reproducibility of candidate screening and sleep improvement by circadian amelioration supported the reliability of these detection criteria. In addition, successful validation of circadian phase estimation at three time points increased the practicality of our method, eliminating the need to interrupt subjects’ sleep for sample collection.

However, several limitations remain. First, more reliable definition of the detection criteria for LCRSWD may require additional rules for subject recruitment and further validation using classified subjects. As an example of the former, although subject's past medical history was self-reported in the present study, some psychological monitoring may be required during subject recruitment because psychological status strongly affects sleep. As an example of the latter, our detection criteria may vary depending on age and/or sex. Second, sleep assessment was based only on PSQI in the present study. Although PSQI is a widely used and highly reliable questionnaire for evaluating sleep problems around the world [33,34], additional sleep evaluation based on objective parameters will further strengthen our conclusions. Further, in addition to self-reporting using MCTQ, habitual sleep timing should be objectively monitored using wearable devices. In particular, sleep debt during work days should be estimated based on total sleep duration because it affects GUf – GUw. Third, improving the accuracy of circadian phase estimation may be required to accelerate practical use. This can be realized by optimizing experimental conditions and identifying additional clock genes whose expression levels are measurable with high accuracy. However, increasing measurements results in increased effort and cost. Fourth, in the present study, we validated our detection criteria for LCRSWD by confirming the reproducibility of candidate screening and sleep improvement by circadian amelioration. Although it took about two months and more than three weeks to perform these respective observations, it may be possible to increase the robustness of the validation by further extending the observation period of these follow-up experiments. Fifth, our present results revealed that the contribution of LCRSWD to sleep problems in the H-PSQI group was unexpectedly small. Only three among 23 subjects met the detection criteria for LCRSWD. The original sample size was therefore insufficient to perform robust follow-up experiments. More extensive trials are required to further validate our detection method for LCRSWD.

## Author contributions

M.A. conceived and supervised the project and wrote the manuscript. R.S., R.M. and A.N. performed experiments. I.T. analysed data and contributed to writing the manuscript. K.N. provided a wide range of general support and gave conceptual advice.

## Funding sources

This work was supported by the Akaeda Medical Research Foundation (M.A.), the Daiwa Securities Health Foundation (R.M.) and the Japan Society for the Promotion of Science (M.A.).

## Declaration of Competing Interest

The authors declare no competing interests.

## Appendix Supplementary materials

Image, image 1

Image, image 2
