# Supplementary figures and images for: A detection method for latent circadian rhythm sleep-wake disorder
Source: eBioMedicine. 2020 Nov 6;62:103080. doi: 10.1016/j.ebiom.2020.103080 (PMC7653065; doi:10.1016/j.ebiom.2020.103080)

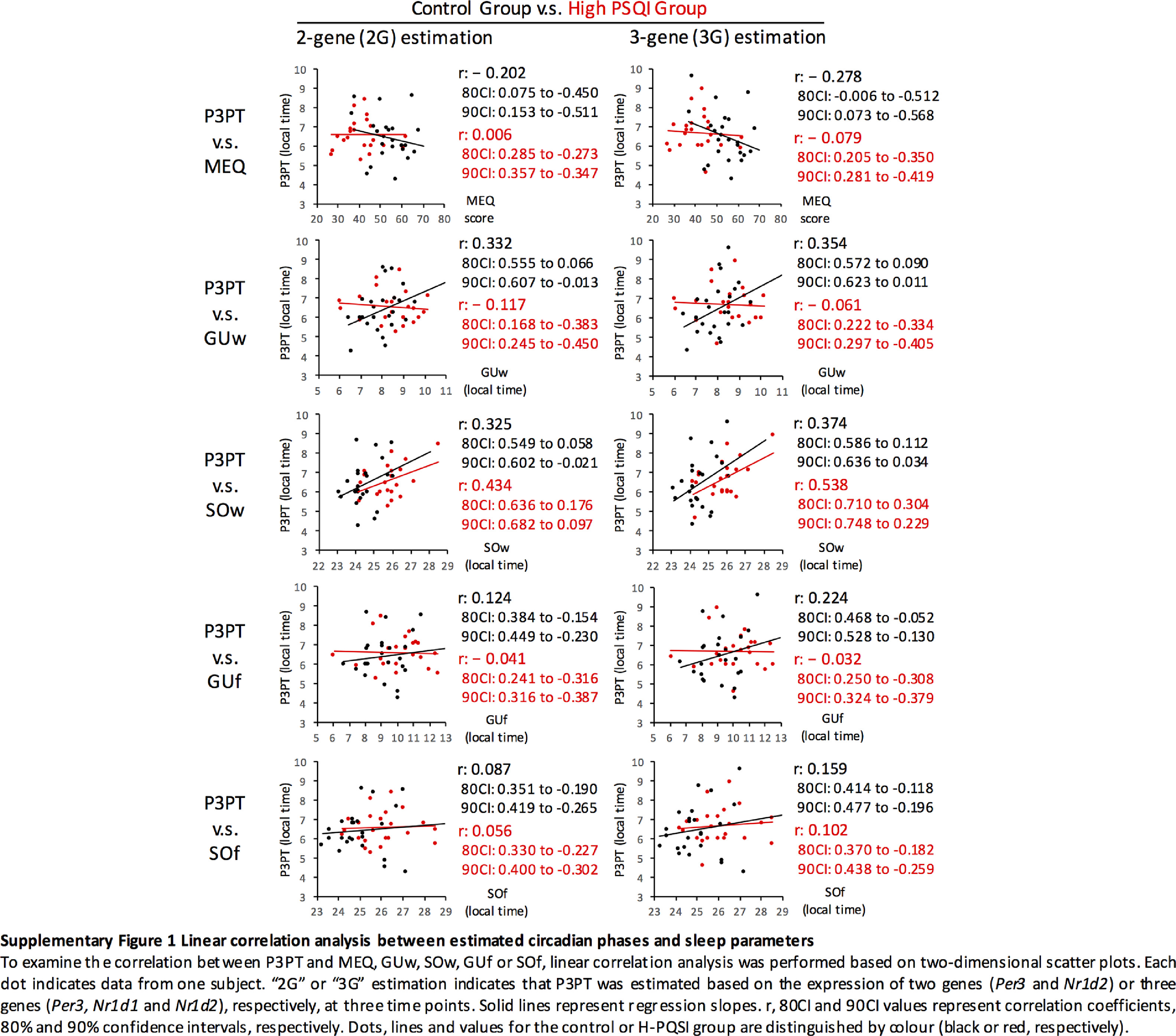

Supplement: Supplementary file 1 [file mmc1.jpg]

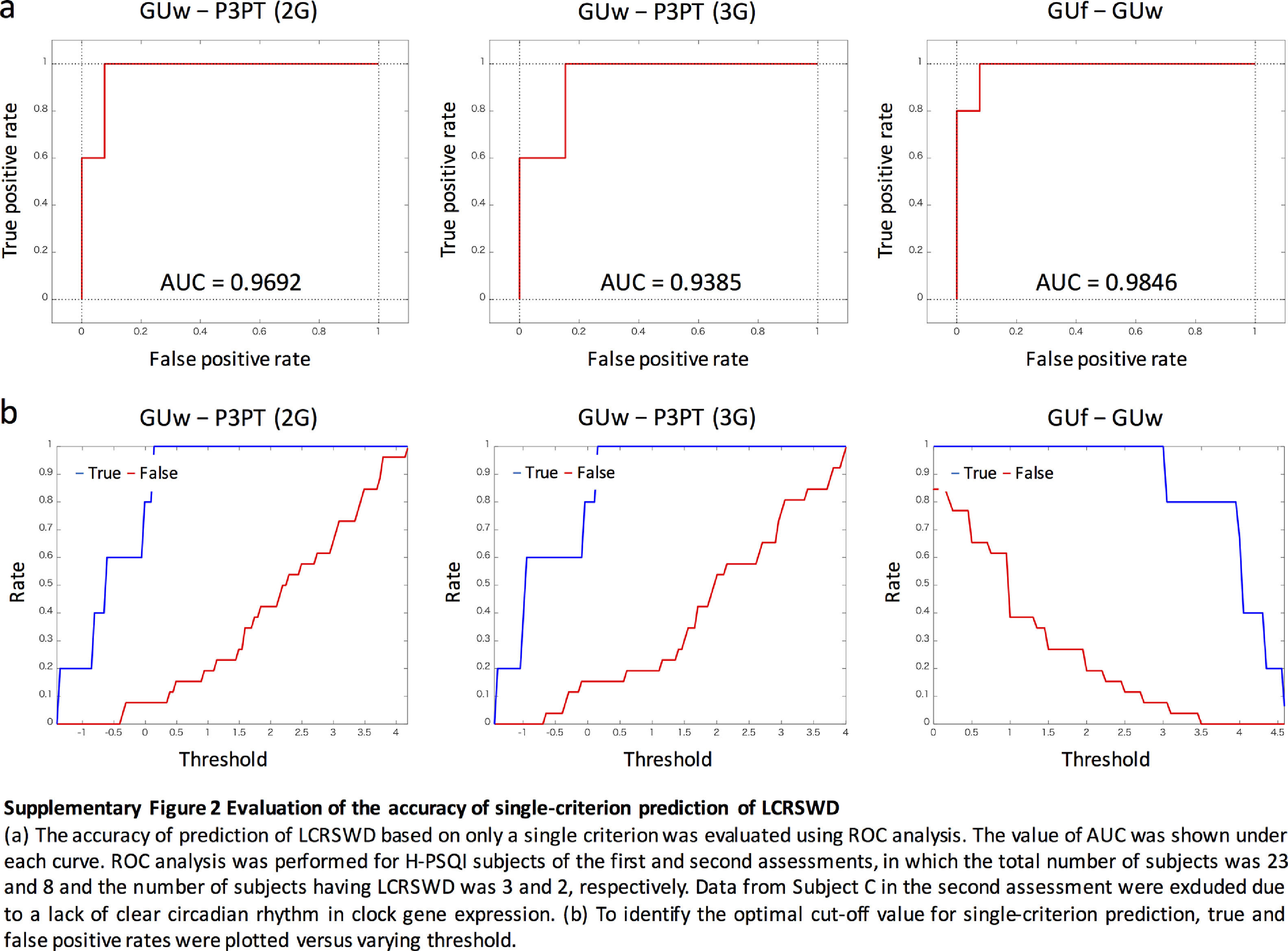

Supplement: Supplementary file 2 [file mmc2.jpg]
